# Thermodynamic Characteristics and Selectivity of the Liquid-Phase Adsorption of Aromatic Compounds on Hypercrosslinked Polystyrene Networks with Ultimate-High Crosslinking Densities by Data of Liquid Chromatography

**DOI:** 10.3390/ijms25031551

**Published:** 2024-01-26

**Authors:** Bulat R. Saifutdinov, Aleksey K. Buryak

**Affiliations:** A.N. Frumkin Institute of Physical Chemistry and Electrochemistry, Russian Academy of Sciences, Building 4, 31 Leninsky Prospect, 119071 Moscow, Russia; akburyak@mail.ru

**Keywords:** hypercrosslinked polystyrene networks (HPSNs) with ultimate crosslinking degrees, liquid-phase adsorption, high-performance liquid chromatography (HPLC), selectivity, separation, thermodynamics

## Abstract

This study delves into the thermodynamics of liquid-phase adsorption on hypercrosslinked polystyrene networks (HPSNs), widely recognized for their distinct structure and properties. Despite the considerable progress in HPSN synthesis and characterization, gaps persist regarding the chromatographic retention mechanism, thermodynamics of adsorption, and their impact on the adsorption selectivity, especially in the case of networks with ultra-high crosslinking densities (up to 500%). Utilizing high-performance liquid chromatography (HPLC), we have explored, for the first time, the thermodynamic intricacies of liquid-phase adsorption onto HPSNs crosslinked in the entire range of the crosslinking degree from 100 to 500%. Our findings reveal the dependences of thermodynamic characteristics and selectivity of adsorption on the crosslinking degree, textural features, and liquid-phase composition in the region of extremely low adsorbent surface coverages (Henry’s range). We have detected that, in the case of HPSNs, the dependence of the thermodynamic characteristics of adsorption on the liquid-phase composition is different than for classical HPLC stationary phases. Moreover, we scrutinize the impact of the molecular structure of the studied aromatic compounds on the thermodynamic characteristics and selectivity of the liquid-phase adsorption on HPSNs. Investigating liquid-phase adsorption selectivity, we demonstrate the pivotal role of π-π interactions in separating aromatic compounds on HPSNs. Eventually, we unveil that the thermodynamic characteristics of adsorption peculiarly depend on the crosslinking degree due to the profound impact of the crosslinking on the electronic density in benzene rings in HPSNs, whereas the separation throughput peaks for the polymer with a 500% crosslinking degree, attributed to its exceptionally rigid network structure, moderate swelling and micropore volume, and minimum specific surface area.

## 1. Introduction

Over the past four decades, high-performance liquid chromatography (HPLC) has emerged as a prevalent, to some extent, one-of-a-kind tool, proving effective in the separation and bioanalysis of diverse mixtures, encompassing both natural and synthetic compounds, including thermolabile ones [1]. Indeed, it is widely utilized in proteomics and other omics technologies, medical studies, environmental monitoring, petroleum chemistry, chemical technology, forensic investigations, etc. Furthermore, in the last 25 years, HPLC has also evolved as one of the key methods for the biophysical and physicochemical characterization of biomolecules and surfaces [2]. Nowadays, most separations by the HPLC technique are carried out primarily using chemically modified silica gels, which have some demerits, such as insufficient chemical stability and lack of selectivity toward some multicomponent mixtures [3]. Therefore, the search for and the investigation of novel cutting-edge porous materials with improved chemical stability and enhanced adsorption selectivity that can be utilized as stationary phases in HPLC continue to be important trends in the physical and analytical chemistry of modern chromatography [4]. Despite current achievements of hyphenated techniques such as liquid chromatography–mass spectrometry, which allow gauging the quantitative and qualitative composition of most mixtures, the availability of selective stationary phases for HPLC is essential to distinguish and determine hard-to-separate components of complex mixtures [5].

In addition to conventionally employed chemically modified silica gels [6] and infrequently utilized porous graphitic carbon [7], contemporary research focuses on state-of-the-art nanoporous three-dimensional adsorbing materials, proposed as HPLC stationary phases, such as metal-organic coordination polymers [8] and hypercrosslinked polymers [9]. Hypercrosslinked polystyrene networks (HPSNs) are considered to be prominent among the latter. Note that these polymeric materials, also recognized as Davankov-type polymers, stand out for their eminent attributes [10]. These include heightened thermal stability, ample chemical inertness, regenerability, microporosity, large specific surface area, concomitantly outstanding adsorption kinetics, homogeneous surface, constant size, and regular spherical shape of particles. As a result of their unique surface chemistry, HPSNs can separate electrolytes and be employed as sorbents upon hemodialysis in blood purification [10]. Notably, HPSNs exhibit elevated selectivity in adsorbing molecules with π-bonds, facilitating the separation of aromatic compounds in complex mixtures, including petroleum fractions and oil refinery products [11,12,13].

**Figure 1:**
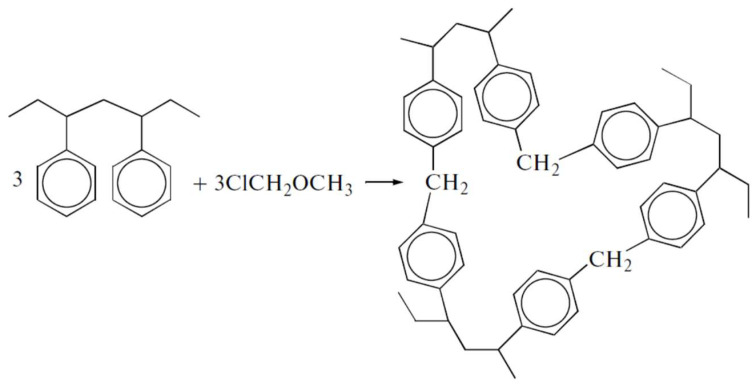
Scheme of the synthesis of hypercrosslinked polystyrene with a crosslinking degree of 100% [20].

Hypercrosslinked polystyrenes (HPSs) are conventionally synthesized through additional crosslinking of styrene-divinylbenzene copolymer chains in a thermodynamically favorable solvent [14]. This results in a three-dimensional polymeric network where each benzene ring is bonded with those of other polystyrene chains at least once or more (Figure 1). However, from the chromatographic point of view, these networks have a disadvantage because they tend to swell in aqueous organic media, commonly used as eluents in HPLC [10,15,16,17,18]. This restricts their application as stationary phases in HPLC due to elevated pressure at the inlet of a chromatographic column. A potential remedy involves further crosslinking to achieve exceptionally high degrees, up to 500%, ensuring virtually all benzene ring positions form chemical bonds. This hypothetical HPS configuration, where all vacant five positions in each benzene ring are bonded with benzene rings of the same or other polymeric chains via methylene bridges, boasts a 500% crosslinking degree and exhibits a resistant, expanded, open three-dimensional network that is resistant to excessive swelling [15,19].

**Figure 2:**
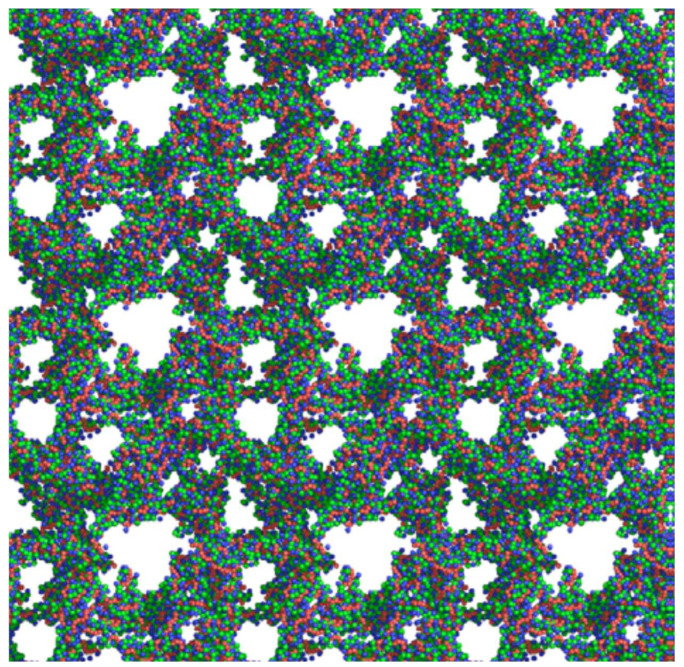
Three-dimensional model of porous structure of hypercrosslinked polystyrene [32].

The physicochemical characteristics, structure, and selective static adsorption of several substances on HPSNs have been thoroughly studied by V.A. Davankov and his colleagues [15,16,17,21,22,23,24,25,26,27]. In particular, structural mobility, surface chemistry, and swelling of HPSNs were investigated via the solid-state NMR, positron annihilation, FT-IR, XPS techniques, etc. Moreover, the three-dimensional structure of HPSNs was studied utilizing molecular dynamics simulations and the quantum chemical approach (Figure 2) [28,29,30]. Nonetheless, to date, to the best of our knowledge, a comprehensive study of the thermodynamics of liquid-phase adsorption across the entire range of crosslinking degrees (100–500%) and in various mobile phase compositions remains unaccomplished. Additionally, the relationship between the thermodynamics of adsorption and separation selectivity has yet to be elucidated. In our opinion, knowledge of the thermodynamic characteristics of adsorption on HPSNs from solutions and their dependence on the crosslinking degree and mobile phase composition is crucial for unraveling the chromatographic retention mechanism and intermolecular interactions in the HPLC system. This understanding facilitates the regulation and optimization of separation conditions by HPLC using the HPS stationary phases [31].

In order to comprehend the adsorption properties of HPSNs under liquid-phase conditions, we selected the HPLC technique. Our prior works [31,33,34,35,36] presented the thermodynamic characteristics of the liquid-phase adsorption of some aromatic and heterocyclic compounds on HPSNs with different crosslinking degrees. In our previous publications [31,34], preliminary research was conducted—particularly, the thermodynamics of the liquid-phase adsorption on the HPSNs crosslinked up to 300–500% was investigated. Subsequently, the effect of the nature of the liquid phase (eluent) on the thermodynamic characteristics of adsorption was also examined [35]. Still, up to date, the thermodynamics of liquid-phase adsorption in the entire range of crosslinking degrees from 100% up to 500% has not yet been described in the literature. Moreover, so far, there are no data on the dependence of the thermodynamic characteristics and selectivity of adsorption on HPSNs with ultimate-high crosslinking degrees on the eluent composition, while these data are essential for optimizing HPLC separations.

Thus, the current study addresses this gap, reporting and providing a detailed analysis of the thermodynamic characteristics and selectivity of the adsorption of some aromatic compounds from aqueous acetonitrile solutions at the wide range of acetonitrile volume fractions from 40 to 70 vol.% onto HPSNs, with crosslinking degrees spanning from 100 to 500%. Herein, for the first time, we report the thermodynamic characteristics of adsorption on HPSNs crosslinked up to 100 and 200%. Then, we compare them with ones previously reported by us for the networks crosslinked up to 300–500% and elucidate the impact of the crosslinking degree and textural characteristics of HPSNs on the thermodynamic characteristics and selectivity of liquid-phase adsorption. We also pay particular attention to investigating the influence of the eluent composition on the thermodynamic characteristics of adsorption and the role of molecular structure in the liquid-phase adsorption on HPSNs with ultimate-high crosslinking degrees.

## 2. Results and Discussion

### 2.1. Morphology and Textural Characteristics

**Figure 3:**
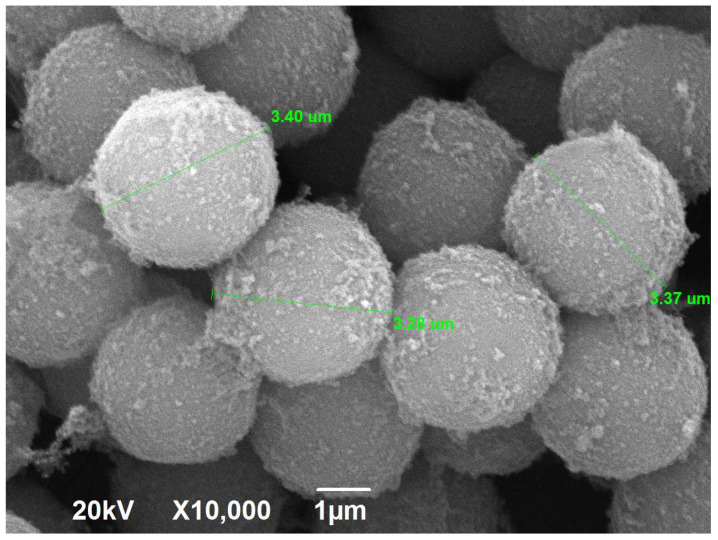
SEM micrograph of the hypercrosslinked polystyrene powder with a crosslinking degree of 100%.

The SEM micrograph of hypercrosslinked polystyrene (HPS) powder with 100% crosslinking is presented in Figure 3. Similar patterns are characteristic of the other HPNs with higher crosslinking densities. The constant size of approximately 3.3 µm and the regular spherical shape of particles are features of the prepared hypercrosslinked polystyrene networks (HPSNs) with ultimate-high crosslinking degrees from 100 to 500%. The primary outcome of the obtained SEM micrographs for high-performance liquid chromatography (HPLC) is that the abovementioned attributes facilitate mass transfer and diffusion throughout the porous layer, as well as adsorption kinetics upon the chromatographic process, and ensure the operation of the synthesized polymeric materials as stationary phases for HPLC [37].

#### 2.1.1. N_2_ and Ar Adsorption at 77 K

Table 1 shows the textural characteristics of HPS powders with different crosslinking degrees *X*, measured by N_2_ adsorption at 77 K, and argon heat desorption techniques. Herein, the textural characteristics for HPS samples with *X* = 100, 200% are provided for the first time and compared with values previously measured by us for polymers with *X* = 300–500% [34]. These data are the evidence of the well-developed microporous structure of all the prepared networks in which the adsorption of molecules occurs. At the same time, the studied HPS stationary phases contain mesopores that facilitate mass transfer throughout the porous layer during the chromatographic process (Table 1) [1].

**Figure 4:**
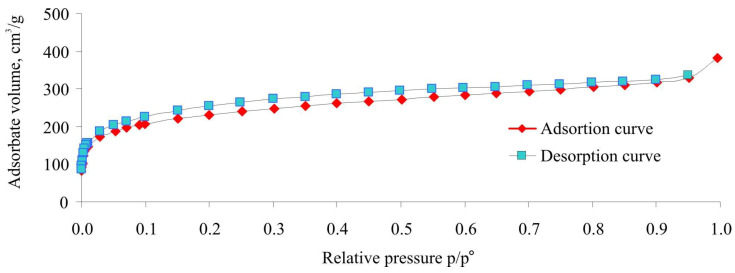
N_2_ adsorption isotherm at 77 K for the hypercrosslinked polystyrene powder with a crosslinking degree of 100%.

The N_2_ adsorption isotherm at 77 K for the HPS powder with a crosslinking degree of 100% is represented in Figure 4. Similar isotherms are characteristics of the HPSs with higher crosslinking degrees up to 500%. The adsorption–desorption hysteresis is observed in a wide range of relative pressures due to the swelling of the HPSNs and is related to their adsorption deformation [38]. Hence, these porous polymeric materials exhibit tremendous adsorption capability toward different substances, even nitrogen and argon molecules.

Note that the Langmuir specific surface area more correctly reflects genuine values of this characteristic in the case of microporous materials than the specific surface area computed in terms of the BET model [39]. Moreover, Langmuir specific surface areas are closer to the corresponding values determined by the argon heat desorption. This is due to the obvious fact that argon penetrates in smaller pores than nitrogen, showing higher values of specific surface area.

As seen in Table 1, the crosslinking degree profoundly impacts the textural characteristics of the studied HPSNs. In fact, the specific surface area gradually changes as the crosslinking increases from 100 to 500%. The maximum value of the specific surface area is characteristic of the HPS sample crosslinked up to 200%, while the network with *X* = 500% has the minimum surface area.

#### 2.1.2. Benzene Vapor Adsorption at 293 K

Table 2 displays the effective textural features of the HPS powders with varying crosslinking degrees (*X*), as measured by the benzene vapor adsorption technique at 293 K. According to the presented data, all HPSNs that were prepared exhibit mature microporous and mesoporous structures, which provide a large cumulative free volume for adsorption and mass transfer throughout the chromatographic column, as well as high adsorption potential. These measurements align with the textural characteristics obtained by nitrogen adsorption at 77 K and argon heat desorption (Table 1).

It should be noted that textural characteristics calculated using equations of the predominantly used adsorption models, such as BET, Langmuir, BJH, and Dubinin–Radushkevich, are apparent parameters because these models do not take into account swelling and adsorption deformation of porous materials [38,39,41]. Meanwhile, according to the data presented in Figure 4 and Table 1 and Table 2, the prepared HPS samples adsorb huge amounts of nitrogen, argon, or benzene vapors and, consequently, exhibit large specific surface areas. Moreover, according to the data presented in Table 2, the maximum micropore volume is characteristic of HPS crosslinked up to 200%. Note that this sample also has the maximum specific surface area and porous volume. At the same time, the polymer with the lowest specific surface area, HPS with *X* = 500%, has moderate values of the micropore volume (Table 2).

### 2.2. Test Adsorbates

Table 3 represents the structural formulae of the studied aromatic compounds numbered **1** to **18**. These compounds were utilized as test adsorbates in the study in order to determine the adsorption properties of the polymeric adsorbents under conditions of HPLC in the range of extremely low adsorbent coverages (so-called Henry’s region). It should be noted that the study of adsorption in Henry’s region enables direct estimating of an adsorbate–adsorbent interaction because there are no lateral interactions in an adsorption layer. The molecular structures were chosen to reveal various types of intermolecular interaction in the liquid-phase adsorption system, which includes HPSNs, and to demonstrate the impact of intramolecular effects on adsorption on the prepared polymeric stationary phases.

### 2.3. Thermodynamic Characteristics of Adsorption on Hypercrosslinked Polystyrenes vs. Crosslinking Degree

#### 2.3.1. Henry Adsorption Constants and Gibbs Energies of Adsorption

Table 4 presents the Henry adsorption constants *K*_1,c_ and standard molar Gibbs energies Δ_a_G* of the adsorption of the studied aromatic compounds (**1**–**18**) from a MeCN–H_2_O (60:40 *v*/*v*) solution on the HPSs with different crosslinking degrees *X* at a temperature of 323 K. Herein, the thermodynamic characteristics of liquid-phase adsorption of the studied aromatic compounds on the HPS samples with *X* = 100, 200% are reported for the first time and compared with values previously measured by us for polymers with *X* = 300–500% [31]. According to the obtained data, liquid-phase adsorption on HPSNs is a spontaneous process. This is because intermolecular interactions between adsorbate molecules and HPSN fragments are stronger than intermolecular interactions between adsorbate and solvent molecules, as well as between the solvent and the polymer. This accounts for the observed spontaneity.

These data indicate that the maximum adsorption is observed for the HPSNs with odd crosslinking degrees. In contrast, for the networks with even crosslinking degrees, there are decreased values of the Henry adsorption constant and modulus of standard molar Gibbs energy of adsorption. Moreover, among the HPSNs with odd crosslinking degrees, the minimum *K*_1,c_ and |Δ_a_G*| values are an attribute of the HPS with a crosslinking degree of 500%. Note that this HPS sample has the minimum specific surface area (Table 1 and Table 2), moderate micropore volume (Table 2), moderate swelling [15,17], and exceptionally rigid network structure [10,19,25]. Expressly, the optimal combination of those parameters facilitates mass transfer throughout a chromatographic column and, consequently, enhances the separation throughput. This is ideal for using HPLC to separate complex mixtures because there is no irreversible or extremely strong adsorption.

The molecular structure of the studied adsorbates significantly impacts the selectivity of the liquid-phase adsorption on HPSNs under HPLC conditions. Indeed, for all the HPSNs, the highest adsorption activity is characteristic of tricyclic aromatic compounds (**11**–**13**). Substances with two aromatic rings in a molecule (**6**, **7**, **9**) exhibit medium adsorption characteristics, while for monocyclic aromatic compounds (**1**, **2**, **4**, **17**), adsorption is not as strong. On the one hand, compounds containing in their molecules center capable of being solvated with components of the MeCN–H_2_O mobile phases (**5**, **8**, **10**, **14**–**16**, **18**) manifest decreased values of *K*_1,c_ and |Δ_a_G*| due to specific intermolecular interaction in the liquid phase. On the other hand, blocking these centers, e.g., by substituting the pyrrole hydrogen with a methyl group, leads to the elevation of the adsorption characteristics as the transition from compound **8** to **9**. Molecules with π-conjugation (**3**) adsorb on the HPSs more potently than the other monocyclic compounds. Remarkably, adding two methyl groups into the molecule of dibenzothiophene (compound **12**) as the transition to 4,6-dimethyldibenzothiophene (compound **13**) drastically strengthens adsorption binding of the heterocycle by HPSNs (Table 3 and Table 4). Obviously, this is because the effect of hyperconjugation increases the electronic density in the aromatic part of the molecule. As a consequence, the energy of π-π-interaction between π-systems of the studied aromatic compounds and π-fragments of HPSNs also possibly increases. To sum up, the longer the chain of π-conjugation in an adsorbate molecule, the stronger its adsorption on HPSNs. This pattern, which is intrinsic for the reversed-phase HPLC variant employed in the current study, is similar to those previously observed for so-called quasi-normal-phase HPLC using HPS stationary phases [11,12].

#### 2.3.2. Enthalpies and Entropies of Adsorption

Table 5 includes the standard molar enthalpies Δ_a_H* and entropies Δ_a_S* of the adsorption of the studied aromatic compounds (**1**–**18**) from a solution made up of 60 vol.% MeCN and 40 vol.% H_2_O onto the HPSs with varying crosslinking degrees (*X*) at a temperature of 323 K. Based on the presented data, it can be concluded that the liquid-phase adsorption on all HPSNs is physical and exothermic. This is due to the adsorbate–adsorbent interaction energy being higher than the sum of energies of intermolecular interaction of adsorbate molecules with a solvent and of solvent molecules with a polymer. Notably, the Δ_a_H* values depend on the molecular structure of the adsorbates in a similar way as the Δ_a_G* ones (Table 4 and Table 5).

It is important to note that the enthalpy does not significantly depend on the crosslinking degree. However, the localization of the adsorbed molecules is stronger on the networks with a crosslinking degree higher than 100%, whereas on the HPS crosslinked by 100%, adsorption occurs primarily due to the energetic factor because the entropy sometimes has even positive values. This is probably because this sample is most swelled and solvated by the molecules of mobile phase components [15]. Hence, ultra-high crosslinking densities can solve issues related to swelling [15].

Thus, as can be seen from the data provided in Table 4 and Table 5, the dependences of the thermodynamic characteristics of the liquid-phase adsorption on the HPSNs samples on the crosslinking degree are not monotonous. Hypothetically, considering the nature of the intermolecular interactions between the polymer matrix and the tested chemicals [10,11,12], the crosslinking impacts the electronic density of benzene rings of the HPS structure. In the networks with *X* = 100, 300, 500%, each benzene ring is substituted two, four, or six times correspondingly, while in the structure of the polymers crosslinked up to 200 and 400%, each benzene ring is substituted three or five times correspondingly. The even number of substituted positions in a benzene ring probably elevates the electronic density, whereas the odd number reduces it. As a result, the π-π-interaction is stronger between adsorbates and networks with the odd crosslinking degrees. The proposed hypothesis is an attempt to shed light on the fact that the HPSNs with the odd crosslinking degrees exhibit elevated values of thermodynamic characteristics of adsorption compared with polymers crosslinked up to even crosslinking degrees.

### 2.4. Thermodynamic Characteristics of Adsorption on Hypercrosslinked Polystyrenes vs. Liquid Phase Composition

#### 2.4.1. Henry Adsorption Constants and Gibbs Energies of Adsorption

Table 6 shows the Henry adsorption constants *K*_1,c_ and standard molar Gibbs energies Δ_a_G* of the adsorption of the studied different aromatic compounds (**1**–**18**) from solutions of varying MeCN–H_2_O compositions (*v*/*v*) onto the HPS sample with a crosslinking degree of *X* = 300% at a temperature of 323 K. The Henry constants of adsorption become notably high when tricyclic aromatic compounds are adsorbed from the MeCN–H_2_O mixture with the highest water content. This is because, in addition to dispersive van der Waals forces and π-π-interaction, hydrophobic expulsion manifests during adsorption from this polar medium onto the nonpolar HPS interface [42]. Furthermore, the *K*_1,c_ and Δ_a_G* values naturally diminish as the volume fraction of MeCN in the mobile phase increases. Intensifying the solvation of adsorbate molecules by the solvent as the organic component concentration increases accounts for the observed pattern described by the Snyder model [43]. This trend is similar to that observed for traditional stationary phases used in HPLC, such as chemically modified silicas and porous graphitic carbon [43,44]. The data presented in Table 6 facilitate optimizing the conditions of HPLC separation utilizing HPSNs.

#### 2.4.2. Enthalpies and Entropies of Adsorption

Here are the standard molar enthalpies Δ_a_H* and entropies Δ_a_S* for the adsorption of the studied aromatic compounds (**1**–**18**) at 323 K, using the HPS sample with a crosslinking degree of *X* = 300% (Table 7). The adsorption was carried out from solutions of different compositions (*v*/*v*) of MeCN–H_2_O. As well known, the participation of a solvent in the liquid-phase adsorption process complicates the chromatographic retention mechanism. It contributes to the thermodynamic characteristics of adsorption because solvates both an adsorbate and adsorbent [1].

In fact, the Gibbs energy of adsorption on the HPSNs depends on the composition of the aqueous organic mobile phase in a similar way as on classical HPLC stationary phases. However, the values of Δ_a_H* and Δ_a_S* (enthalpy and entropy changes, respectively) increase in absolute value with the rise of the MeCN volume fraction in the eluent. This observed pattern is unusual for HPLC [43,44]. The most substantial adsorption binding and maximum localization of adsorbed molecules in the polymeric matrix occur when the adsorption is from the MeCN–H_2_O mixture with the highest content of the organic modifier. In our opinion, the different nature of polymer swelling, depending on the water and MeCN content in the liquid phase, is a possible account for the observed pattern.

### 2.5. Structural Selectivity of Hypercrosslinked Polystyrenes and Retention Mechanism

**Figure 5:**
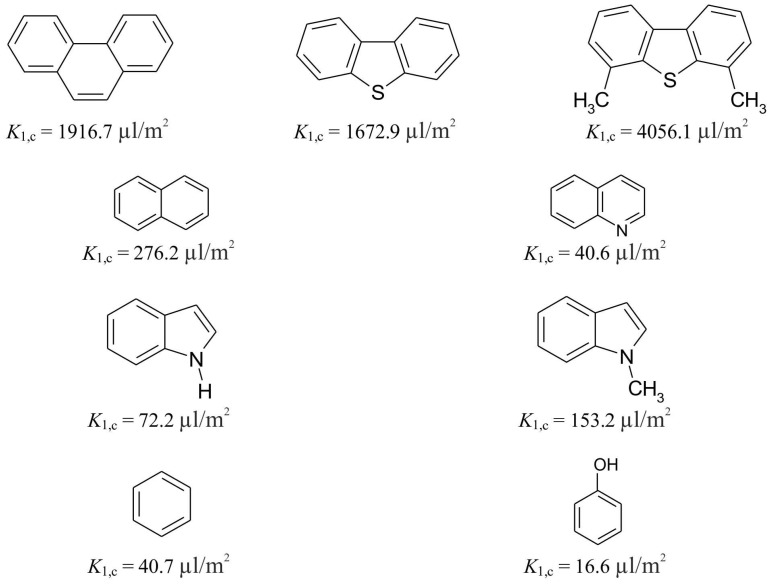
Structural selectivity of the hypercrosslinked polystyrene sample with a crosslinking degree of 500% toward some aromatic compounds upon their adsorption from the MeOH–H_2_O (60:40 *v*/*v*) solution.

Figure 5 demonstrates the adsorption selectivity of the prepared HPS with a 500% crosslinking degree toward certain aromatic compounds upon adsorption from a solution of MeOH–H_2_O (60:40 *v*/*v*). The more aromatic rings in the molecule, the stronger the adsorption of the studied compounds on the HPSs. Indeed, the Henry adsorption constant increases significantly as more aromatic rings are added to an adsorbate molecule. Moreover, adding two methyl groups into the dibenzothiophene molecule greatly elevates the *K*_1,c_ value. This is probably due to the increased strength of the π-π interaction, which is caused by the positive inductive effect of the two methyl groups, as well as the impact of hyperconjugation and the resulting increase in the electronic density on the aromatic part of the molecule. As a result, the π-system of 4,6-dimethyldibenzothiophene molecules stronger interacts with the π-systems of the HPSNs through the π-π interaction. On the contrary, if the adsorbate molecule contains a pyridine nitrogen atom or a pyrrole acid center, the adsorption of aromatic compounds in the liquid phase is reduced due to their solvation by polar solvent components. Thus, retention upon reversed-phase HPLC on HPSNs is carried out through dispersive van der Waals forces, π-π interaction, and hydrophobic expulsion with the pivotal role of π-π interaction in the adsorption selectivity.

### 2.6. Selectivity and Throughput of HPLC Separation by Hypercrosslinked Polystyrenes

**Figure 6:**
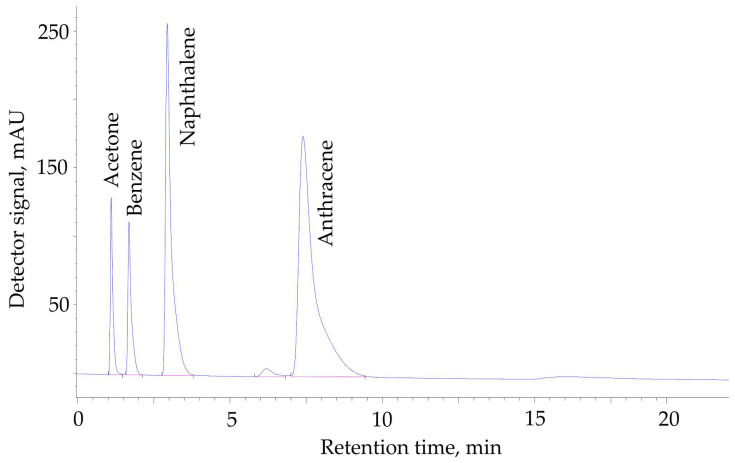
HPLC separation of acetone, benzene, naphthalene, and anthracene using the chromatographic column (50 mm × 3 mm) packed by the hypercrosslinked polystyrene with a crosslinking degree of *X* = 500%. A mobile phase is a MeCN–H_2_O (90:10 *v*/*v*) mixture. A flow rate is 0.3 mL/min.

The chromatographic columns packed with the HPS powders having a crosslinking degree of *X* = 100–500% were used to separate acetone, benzene, naphthalene, and anthracene utilizing HPLC. Figure 6 depicts the typical chromatogram obtained during the separation on the HPS with a 500% crosslinking degree. It should be noted that the order of the release of analytes from a chromatographic column on chromatograms registered upon HPLC using the HPS samples as stationary phases, which have a varied crosslinking degree from 100 to 500%, remains constant. This indicates that the polymeric stationary phases prepared have similar adsorption selectivity, and no size-exclusion chromatographic effects were detected (Table 4).

Additionally, we observed nearly Gaussian peaks in the chromatogram. Such shape of the peaks was caused by the regular spherical shape of HPS particles and the presence of transport mesopores in the HPSN structure, which enhanced mass transfer, diffusion, and adsorption kinetics. Large surface area and strong π-π interaction account for some peak distortion in the case of the bicyclic and tricyclic aromatic compounds. Although the crosslinking degree does not affect adsorption selectivity, the highest HPLC separation throughput can be achieved using the HPSN, which is crosslinked up to 500%. This is possibly due to the optimal rigid structure of this network [19,25], its resistance to swelling, unlike HPSs, which are crosslinked until 100–400% [15], as well as the minimum specific surface area and moderate micropore volume (Table 1 and Table 2).

## 3. Materials and Methods

### 3.1. Synthesis of Hypercrosslinked Polystyrenes

All reagents and solvents employed were commercial products (Acros Organics, Antwerpen, Belgium).

Monosized styrene–1% divinylbenzene copolymer microbeads were prepared using the precipitation polymerization technique [45]. The solution of 25 mL styrene, 0.5 mL 65% divinylbenzene, and 0.46 g AIBN was added to the mixture of 63 mL polydiethylsiloxane liquid and 63 mL *n*-octane. All the components were rigorously agitated till obtaining a transparent solution and then heated at 70 °C within 4 h. As this time passed, the reaction mixture was allowed to stand for 20 h at room temperature. The resulting beads of 3.2–3.3 µm in diameter were filtered, rinsed with hexane and ethanol, and subsequently dried at room temperature when necessary.

Monosized microbeads of the above polymer were then additionally crosslinked with 0.5, 1.0, 1.5, 2.0, and 2.5 mole monochlorodimethyl ether per styrene repeating in it. Thus, obtained hypercrosslinked products exhibited the crosslinking degree of 100%, 200%, 300%, 400%, and 500%, respectively. All details of the syntheses were published elsewhere [14,15,19].

The prepared hypercrosslinked polystyrenes (HPSs) with ultimate crosslinking densities have been synthesized at the Nesmeyanov Institute of Organoelement Compounds of the Russian Academy of Sciences (INEOS RAS, Moscow, Russia) in the Prof. Dr. V.A. Davankov laboratory and provided to us for physicochemical characterization and chromatographic studies.

### 3.2. Characterization of Hypercrosslinked Polystyrenes

The HPS samples employed as stationary phases in the current study have been thoroughly and comprehensively characterized in the laboratory of Prof. Dr. V.A. Davankov (INEOS RAS, Moscow, Russia) utilizing different techniques in order to investigate their structure, porous characteristics, and morphology. Their characterization was previously described in detail in [15,19,21,22,26,27,28,31,34].

### 3.3. Chromatographic Measurements

Packaging of the polymer material in steel columns for high-performance liquid chromatography (HPLC) was carried out as follows. The suspension was prepared by dissolving 1.5–2 g of dry polymer in 50% aqueous acetone or ethanol in a measuring cylinder (100 mL). The suspension was subjected to ultrasound treatment for 1–2 min to remove lumps. The suspension was allowed to settle, and the top layer was drained. Then, the solvent was added again and re-treated with ultrasound. The resulting suspension was poured into a special cylindrical tank. At the entrance of this tank, pressure was applied from a pneumatic pump (Alltech, Deerfield, IL, USA), and a steel column was at the exit. When packing the column, the pressure in the system was maintained in the area of 150–200 bar, based on the pressure gauge of the air pump. After spilling two volumes of a cylindrical tank (200 mL) through the system, the packaging was stopped, the pressure was fully relieved, and the steel column was unscrewed. The excess polymeric adsorbent was removed with a special spatula; the column was closed with an end fitting and placed in a liquid chromatograph for washing. Washing with acetonitrile was carried out within 2–3 h. Packing of the chromatographic columns was implemented by Dr. M.M. Ilyin (INEOS RAS).

The studied aromatic compounds used as test adsorbates (Table 3) were commercial products of analytical standard grade (Sigma-Aldrich, St. Louis, MO, USA). Acetonitrile (MeCN) employed as a component of mobile phases was HPLC grade (Panreac, Barcelona, Spain).

The chromatographic equipment, procedure of measuring the liquid-phase adsorption of the studied aromatic compounds utilizing the HPLC technique, and formulae used to calculate the thermodynamic characteristics of adsorption have been described in detail in our previous work [31].

## 4. Conclusions

To sum up, in this study, we report and compare, for the first time, thermodynamic characteristics of liquid-phase adsorption on hypercrosslinked polystyrene networks crosslinked at ultra-high degrees ranging from 100 to 500%. It is evident from our report that all prepared polymers demonstrated constant-size regular spherical particles and a large surface area, developed microporosity, and simultaneously transport mesopores. It was revealed that the thermodynamic characteristics of adsorption peculiarly depend on the crosslinking degree due to the profound impact of the crosslinking on the electronic density in benzene rings in a network, whereas the separation throughput peaks for the polymer with a 500% crosslinking degree, attributed to its exceptionally rigid network structure, moderate swelling and micropore volume, and minimum specific surface area. Overall, all the prepared hypercrosslinked polystyrenes show heightened structural selectivity toward aromatic compounds, emphasizing the pivotal role of π-π interaction in adsorption binding. At last, the current study provides valuable insights into the retention mechanism in high-performance liquid chromatography using hypercrosslinked polystyrenes with ultra-high crosslinking densities. We suggest the use of the presented thermodynamic data to optimize and regulate separation conditions in high-performance liquid chromatography utilizing hypercrosslinked polystyrenes as stationary phases.

## Figures and Tables

**Table 1:** Textural characteristics of the hypercrosslinked polystyrene powders with the different crosslinking degrees *X*, measured by the N_2_ adsorption at 77 K, and argon heat desorption techniques.

Textural Characteristics	*X* = 100%	*X* = 200%	*X* = 300%	*X* = 400%	*X* = 500%
*S*N_2mic_, ^a^ m^2^/g	953	1044	811	1015	673
*S*_Ar_, ^b^ m^2^/g	1050	1554	880	1100	684
*S*N_2_(Langmuir), ^c^ m^2^/g	1101	1189	873	1083	730
*S*N_2_(BET), ^d^ m^2^/g	765	829	605	751	509
*r*(BJH), ^e^ nm	1.5	1.5	1.5	1.7	1.7

^a^ *S*N_2mic_ is the micropore specific surface area; ^b^ *S*_Ar_(BET) is the specific surface area measured by the argon heat desorption; ^c^ *S*N_2_(Langmuir) is the Langmuir specific surface area; ^d^ *S*N_2_(BET) is the BET specific surface area; ^e^ *r*(BJH) is the average pore radius calculated by the BJH model.

**Table 2:** Textural characteristics of the hypercrosslinked polystyrene powders with the different crosslinking degrees *X* measured by the benzene vapors adsorption at 293 K technique.

Textural Characteristics	*X* = 100%	*X* = 200%	*X* = 300%	*X* = 400%	*X* = 500%
*W*_0_, ^a^ cm^3^/g	0.260	0.300	0.232	0.272	0.242
*E*_0_, ^b^ kJ/mole	12.13	14.03	12.92	12.53	12.08
*x*_0_, ^c^ nm	0.82	0.71	0.77	0.80	0.83
*V*_mes_, ^d^ cm^3^/g	0.600	0.580	0.435	0.382	0.409
*V*_S_, ^e^ cm^3^/g	0.860	0.880	0.667	0.657	0.651
*S*_mes_(BET), ^f^ m^2^/g	980	1030	720	770	640
*S*_mes_(γ), ^g^ m^2^/g	330	400	315	320	370

^a, b, c^ *W*_0_, *E*_0_, *x*_0_ are the parameters of the Dubinin–Radushkevich equation [40]: the specific micropore volume, characteristic energy of the benzene standard vapor, and half-width of the micropores, respectively; ^d^ *V*_mes_ is the specific mesopore volume (*V*_mes_ = *V*_S_ − *W*_0_); ^e^ *V*_S_ is the ultimate adsorption volume corresponding to the benzene vapor pressure of *p*/*p_s_* = 1; ^f^ *S*_mes_(BET), ^g^ *S*_mes_(γ) are the specific mesopore surface area calculated utilizing the BET equation the γ-method, respectively.

**Table 3:** Structural formulae of the studied aromatic compounds (**1**–**18**) used as test adsorbates.

No.	Structural Formula	No.	Structural Formula	No.	Structural Formula
**1**	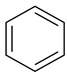 Benzene	**7**	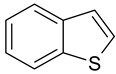 Benzothiophene	**13**	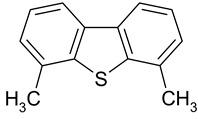 4,6-Dimethyldibenzothiophene
**2**	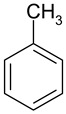 Toluene	**8**	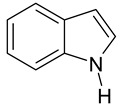 Indole	**14**	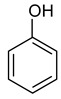 Phenol
**3**	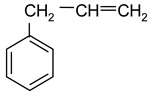 Allylbenzene	**9**	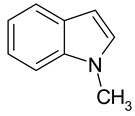 1-Methylindole	**15**	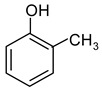 *o*-Cresol
**4**	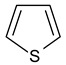 Thiophene	**10**	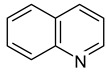 Quinoline	**16**	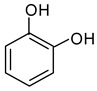 Catechol
**5**	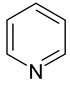 Pyridine	**11**	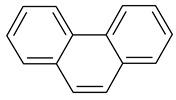 Phenantrene	**17**	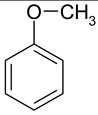 Anisole
**6**	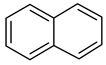 Naphthalene	**12**	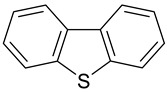 Dibenzothiophene	**18**	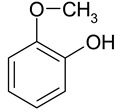 Guaiacol

**Table 4:** Henry adsorption constants (*K*_1,c_, μL/m^2^) and standard molar Gibbs energies (Δ_a_G*, kJ/mole) of the adsorption of the studied aromatic compounds (**1**–**18**) from the MeCN–H_2_O (60:40 *v*/*v*) solution on the hypercrosslinked polystyrenes with the different crosslinking degrees *X* at 323 K.

No.	*X* = 100%		*X* = 200%		*X* = 300%		*X* = 400%		*X* = 500%	
	*K*_1,c_	−Δ_a_G*	*K*_1,c_	−Δ_a_G*	*K*_1,c_	−Δ_a_G*	*K*_1,c_	−Δ_a_G*	*K*_1,c_	−Δ_a_G*
**1**	11.9	6.7	6.7	5.1	12.2	6.7	7.8	5.5	11.7	6.6
**2**	17.2	7.6	9.4	6.0	17.2	7.6	10.8	6.4	15.8	7.4
**3**	62.4	11.1	34.1	9.5	63.6	11.1	38.1	9.8	51.4	10.6
**4**	10.5	6.3	6.0	4.8	11.3	6.5	7.4	5.4	11.1	6.5
**5**	2.2	2.1	1.5	1.2	3.8	3.6	2.0	1.8	3.2	3.1
**6**	37.1	9.7	20.6	8.1	39.6	9.9	24.3	8.6	34.5	9.5
**7**	33.1	9.4	18.5	7.8	36.2	9.6	22.4	8.3	32.2	9.3
**8**	11.3	6.5	7.0	5.2	14.3	7.1	8.8	5.9	13.6	7.0
**9**	23.3	8.5	13.0	6.9	24.7	8.6	15.1	7.3	22.2	8.3
**10**	7.3	5.3	4.8	4.2	11.3	6.5	6.1	4.9	9.1	5.9
**11**	122.3	12.9	68.9	11.4	135.7	13.2	78.6	11.7	106.0	12.5
**12**	116.8	12.8	66.0	11.2	129.4	13.1	76.4	11.6	103.4	12.5
**13**	242.0	14.7	127.2	13.0	237.2	14.7	131.8	13.1	167.7	13.8
**14**	3.6	3.5	2.5	2.4	5.0	4.3	3.3	3.2	5.1	4.4
**15**	5.7	4.7	3.7	3.5	7.5	5.4	4.8	4.2	7.1	5.3
**16**	1.5	1.1	0.9	–0.2	1.6	1.3	1.4	0.9	1.8	1.5
**17**	13.0	6.9	7.2	5.3	13.7	7.0	8.5	5.7	12.6	6.8
**18**	5.5	4.6	3.1	3.1	6.2	4.9	4.4	4.0	6.2	4.9

**Table 5:** Standard molar enthalpies (Δ_a_H*, kJ/mole) and entropies (Δ_a_S*, J/(mole K)) of the adsorption of the studied aromatic compounds (**1**–**18**) from the MeCN–H_2_O (60:40 *v*/*v*) solution on the hypercrosslinked polystyrenes with the different crosslinking degrees *X* at 323 K.

No.	*X* = 100%		*X* = 200%		*X* = 300%		*X* = 400%		*X* = 500%	
	−Δ_a_H*	−Δ_a_S*	−Δ_a_H*	−Δ_a_S*	−Δ_a_H*	−Δ_a_S*	−Δ_a_H*	−Δ_a_S*	−Δ_a_H*	−Δ_a_S*
**1**	8.2	4.84	8.4	10.3	9.7	9.2	8.6	9.4	9.1	7.7
**2**	8.7	3.4	9.1	9.6	10.6	9.2	9.3	9.0	9.8	7.3
**3**	12.4	4.0	13.2	11.7	13.9	8.6	13.3	11.0	13.4	8.9
**4**	8.3	6.2	8.6	11.7	9.5	9.4	8.5	9.8	8.7	7.0
**5**	–	–	–	–	8.9	16.5	–	–	–	–
**6**	10.9	–3.6	11.5	10.5	12.1	6.8	11.4	8.7	11.9	7.5
**7**	10.7	–4.0	11.2	10.4	13.7	12.4	11.2	8.8	11.5	6.7
**8**	9.8	–10.2	10.5	16.3	13.1	18.4	10.7	14.9	10.7	11.5
**9**	10.1	–5.0	10.6	11.6	12.5	12.1	10.4	9.8	12.2	12.1
**10**	2.7	–8.2	3.2	–3.1	8.2	5.4	4.5	–1.2	5.5	–1.2
**11**	14.0	3.5	14.8	10.6	16.4	9.9	14.5	8.6	15.4	8.8
**12**	13.6	2.6	14.5	10.2	16.1	9.5	14.9	10.0	15.0	7.9
**13**	14.5	–0.7	15.4	7.4	16.5	5.5	15.7	8.2	14.3	1.7
**14**	5.5	6.5	6.4	12.3	9.2	15.3	8.0	15.0	6.4	6.3
**15**	6.4	5.2	7.3	11.8	9.8	13.6	8.2	12.3	7.0	5.2
**16**	8.7	–	2.5	8.3	–	–	–	–	–	–
**17**	8.4	4.8	8.8	10.7	10.8	11.7	9.5	11.6	8.4	5.0
**18**	7.0	7.5	5.3	6.8	7.8	8.9	8.7	14.5	6.0	3.4

**Table 6:** Henry adsorption constants (*K*_1,c_, μL/m^2^) and standard molar Gibbs energies (Δ_a_G*, kJ/mole) of the adsorption of the studied aromatic compounds (**1**–**18**) from the MeCN–H_2_O solutions of different composition (*v*/*v*) on the hypercrosslinked polystyrene sample with a crosslinking degree of *X* = 300% at 323 K.

No.	40:60		50:50		60:40		70:30	
	*K*_1,c_	−Δ_a_G*	*K*_1,c_	−Δ_a_G*	*K*_1,c_	−Δ_a_G*	*K*_1,c_	−Δ_a_G*
**1**	41.9	10.0	21.0	8.2	12.2	6.7	7.4	5.4
**2**	71.5	11.5	31.8	9.3	17.2	7.6	9.8	6.1
**3**	431.8	16.3	142.0	13.3	63.6	11.1	31.2	9.2
**4**	34.9	9.5	18.6	7.9	11.3	6.5	7.2	5.3
**5**	6.5	5.0	4.5	4.0	3.8	3.6	3.1	3.0
**6**	206.5	14.3	78.9	11.7	39.6	9.9	21.2	8.2
**7**	183.1	14.0	70.0	11.4	36.2	9.6	19.9	8.0
**8**	57.9	10.9	25.2	8.7	14.3	7.1	8.4	5.7
**9**	119.6	12.8	46.8	10.3	24.7	8.6	13.6	7.0
**10**	30.9	9.2	16.3	7.5	11.3	6.5	8.1	5.6
**11**	1011.8	18.6	309.3	15.4	135.7	13.2	64.8	11.2
**12**	933.6	18.4	290.3	15.2	129.4	13.1	62.7	11.1
**13**	2366.6	20.9	598.8	17.2	237.2	14.7	103.5	12.5
**14**	13.5	7.0	7.3	5.3	5.0	4.3	3.3	3.2
**15**	24.8	8.6	12.0	6.7	7.5	5.4	4.7	4.1
**16**	2.8	2.8	1.8	1.7	1.6	1.3	1.3	0.6
**17**	51.7	10.6	23.3	8.5	13.7	7.0	8.0	5.6
**18**	17.6	7.7	9.3	6.0	6.2	4.9	4.2	3.9

**Table 7:** Standard molar enthalpies (Δ_a_H*, kJ/mole) and entropies (Δ_a_S*, J/(mole K)) of the adsorption of the studied aromatic compounds (**1**–**18**) from the MeCN–H_2_O solutions of different composition (vol./vol.) on the hypercrosslinked polystyrene sample with a crosslinking degree of *X* = 300% at 323 K.

No.	40:60		50:50		60:40		70:30	
	−Δ_a_H*	−Δ_a_S*	−Δ_a_H*	−Δ_a_S*	−Δ_a_H*	−Δ_a_S*	−Δ_a_H*	−Δ_a_S*
**1**	7.1	–9.1	9.0	2.6	9.7	9.2	9.4	12.4
**2**	7.0	–13.7	9.3	0.2	10.6	9.2	10.3	12.9
**3**	11.2	–15.6	13.6	0.9	13.9	8.6	14.2	15.5
**4**	7.7	–5.6	9.3	4.4	9.5	9.4	9.2	12.2
**5**	–	–	–	–	8.9	16.5	–	–
**6**	9.1	–16.2	11.9	0.6	12.1	6.8	12.2	12.3
**7**	–	–	11.6	0.6	13.7	12.4	11.9	11.9
**8**	–	–	11.8	9.9	13.1	18.4	10.4	14.3
**9**	–	–	11.4	3.4	12.5	12.1	10.7	11.4
**10**	–	–	5.7	–5.7	8.2	5.4	8.2	7.9
**11**	11.8	–20.8	14.4	–3.0	16.4	9.9	16.0	14.8
**12**	12.1	–19.3	14.4	–2.7	16.1	9.5	15.7	14.2
**13**	12.6	–25.5	15.3	–5.7	16.5	5.5	16.6	13.0
**14**	–	–	7.8	7.6	9.2	15.3	7.5	13.2
**15**	–	–	8.2	4.7	9.8	13.6	8.2	12.5
**16**	0.4	–7.4	0.0	–5.1	–	–	–	–
**17**	–	–	9.4	2.9	10.8	11.7	8.6	9.2
**18**	–	–	6.2	0.6	7.8	8.9	6.9	9.2

## Data Availability

The data presented in this study are available on request from the corresponding author.
